# Neurostimulation for Stroke Rehabilitation

**DOI:** 10.3389/fnins.2021.649459

**Published:** 2021-05-14

**Authors:** Windsor Kwan-Chun Ting, Faïza Abdou-Rahaman Fadul, Shirley Fecteau, Christian Ethier

**Affiliations:** Département de Psychiatrie et de Neurosciences, Centre de Recherche CERVO, Université Laval, Québec City, QC, Canada

**Keywords:** neurostimulation, neuromodulation, stroke, closed-loop stimulation, optogenetic stimulation, brain-computer interfaces, neural plasticity

## Abstract

Neurological injuries such as strokes can lead to important loss in motor function. Thanks to neuronal plasticity, some of the lost functionality may be recovered over time. However, the recovery process is often slow and incomplete, despite the most effective conventional rehabilitation therapies. As we improve our understanding of the rules governing activity-dependent plasticity, neuromodulation interventions are being developed to harness neural plasticity to achieve faster and more complete recovery. Here, we review the principles underlying stimulation-driven plasticity as well as the most commonly used stimulation techniques and approaches. We argue that increased spatiotemporal precision is an important factor to improve the efficacy of neurostimulation and drive a more useful neuronal reorganization. Consequently, closed-loop systems and optogenetic stimulation hold theoretical promise as interventions to promote brain repair after stroke.

## Introduction

Stroke often leads to neuronal death and permanent dysfunction. It can cause substantial damage to the motor cortex, hinder motor control, and result in a decreased autonomy and quality of life. Through neural plasticity, the brain has the capability to reorganize by forming new connections among residual neurons, which may compensate at least in part for the lost ones. There is a critical time window of enhanced plasticity for 1–3 months after ischemic stroke, during which both spontaneous and intervention-mediated recovery is maximized (Zeiler and Krakauer, 2013). However, spontaneous reorganization is often maladaptive or insufficient to restore function to pre-insult levels. Building evidence suggests an important role for the relative timing of perisynaptic neuronal activity to drive plasticity (Feldman, 2012). Through neurostimulation, it is possible to induce a causal timing between the firing of two neurons and thereby induce Hebbian spike-timing-dependent plasticity (Markram et al., 1997; Bi and Poo, 1998). For this reason, a growing number of researchers are investigating different neurostimulation approaches with the goal of inducing targeted plastic changes in the nervous system to reduce the consequences of lesions and improve function.

Here, we review the main stimulation approaches and techniques whichare being investigated, beginning with an examination of the general principles of stimulation-driven plasticity. Due to the complexity and heterogeneity of nervous system organization, we argue that more targeted stimulation techniques could be more effective in inducing plasticity, and could also result in a neural reorganization with greater functional benefits. Closed-loop stimulation paradigms have an important advantage in this regard, as they rely (at least for the presynaptic component) on naturally occurring patterns of brain activity. Therefore, this approach targets more specifically the neurons involved in voluntary motor control (Ethier et al., 2015). Optogenetic stimulation also has great potential as a tool to stimulate neurons selectively and induce synaptic changes in targeted neuronal subpopulations. This factor could be critical to improving the functional relevance of induced neural plasticity. Its eventual use in humans would pose important practical challenges and require new ethical frameworks. In the meantime, the use of optogenetic and electrical stimulation in animal models will advance our understanding of neural plasticity and recovery mechanisms. We focus on stroke in this review since it is one of the leading causes of death and disability worldwide and current treatments remain scarce. However, the principles of neuronal plasticity discussed, and the new methods available to understand and reshape neuronal connections could theoretically be applied with the appropriate modifications to other types of neurological or psychiatric conditions, providing a unified framework for treatment of brain disease and injury.

## Principles of Stimulation-Driven Plasticity

The repeated coincidence of postsynaptic action potentials with synaptic inputs is the driving factor explaining activity-dependent synaptic changes. Regrouped under the umbrella concept of spike-timing-dependent plasticity (STDP) (Markram et al., 1997; Froemke and Dan, 2002; Dan and Poo, 2004; Feldman, 2012), the exact timing rules by which coincident activity can encourage long-term potentiation (LTP) or depression (LTD) may vary according to the type of neuron, or even synaptic location on the dendritic tree (Froemke et al., 2010). Generally, stimulation interventions designed to leverage STDP to potentiate cortical or corticospinal interconnections aim at increasing the coincidence of action potentials in pre- and postsynaptic neuronal populations with a positive sequential timing (pre before post) in accordance to the Hebbian principles of causal association (Hebb, 1949). In this framework, synapses are strengthened when they repeatedly take part in the generation of action potentials in the postsynaptic neuron, and weakened when they do not.

## Stimulation Paradigms

**FIGURE 1:**
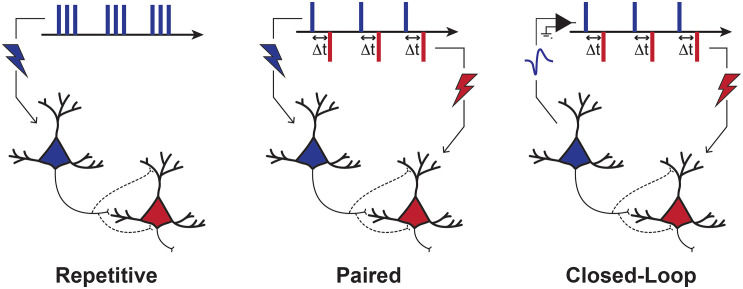
Stimulation Paradigms to Induce Plasticity. Depiction of three main stimulation strategies used to induce plasticity. With repetitive stimulation, pre-synaptic neurons are activated directly and post-synaptic neurons are activated transsynaptically. With paired stimulation, the synchronization of pre- and post-synaptic populations is controlled directly by the stimulation of two points of the nervous system. Repetitive and paired stimulation can be applied in an open-loop manner, with no regard to current brain or behavioral state. In closed-loop approaches, however, the stimulation of a neuronal (post-synaptic) population can be precisely timed with neuronal activity (e.g., action potentials or EEG). These three different strategies all aim to induce coincidental activity in pre- and post-synaptic neurons with such a timing that would result in either LTP- or LTD-like effects, according to the rules of Hebbian STDP.

Three main stimulation approaches may be employed to exogeneously enhance the coactivation of connected neurons: (1) repetitive stimulation, (2) paired stimulation, and (3) closed-loop stimulation (Jackson and Zimmermann, 2012; Figure 1). Repetitive stimulation can recruit presynaptic neurons directly and postsynaptic neurons transsynaptically if the interconnecting synapses allow it. With paired stimulation, a precise relative timing of activity can be imposed to two interconnected neuronal populations by applying stimulation pulses at two distinct locations within the nervous system. Closed-loop stimulation involves a more sophisticated stimulation control system, which synchronizes the stimulation of a postsynaptic neuronal population with spontaneous activity in presynaptic neurons, detected from neuronal recordings or brain imagery. In some other forms, closed-loop stimulation may involve synchronizing the stimulation on muscular or behavioral activity instead of neuronal recordings (e.g., stimulation triggered by EMG signals, movement detection or task events). These three general strategies are not mutually exclusive. For example, Zrenner et al. (2018) have recently shown that the LTP-like effect of repetitive stimulation could be improved when it’s applied precisely during a particular brain state (i.e., negative phase of μ-rhythm).

In the following sections, we will describe different neurostimulation approaches based on these three stimulation paradigms. Although other stimulation techniques exist, we will focus our analysis on experiments that have employed transcranial magnetic stimulation (TMS), electrical stimulation and optogenetic stimulation approaches, the three major types of neurostimulation currently employed in the clinical and fundamental neurosciences today.

## Repetitive Stimulation

### Transcranial Magnetic Stimulation (TMS)

TMS in humans has been widely used on the primary motor cortex to elicit descending corticospinal volleys and muscle contraction (TMS-induced motor evoked potentials, MEPs). This non-invasive neurostimulation technique has been applied using a number of different protocols, with the goal of altering corticomotor excitability toward stroke recovery. Repetitive TMS (rTMS) and theta burst stimulation (TBS) paradigms are the most common TMS applications aimed at driving plasticity based on the repetitive stimulation paradigm shown in Figure 1. Low (≤1 Hz) and high (≥5 Hz) frequency rTMS are generally employed with the premises that they respectively produce a decrease and an increase in excitability of stimulated regions respectively (Fitzgerald et al., 2006). The TBS paradigm, consisting of bursts of three pulses at 50 Hz, delivered every 200 ms, can also be used to modulate excitability in either direction. Trains of bursts can be delivered continuously for 20–40 s (cTBS), typically resulting in a depressive effect on cortical excitability, or intermittently (iTBS), by applying TBS trains for only 2 s at a time with 8 s pauses in between, generally leading to facilitatory effects (Cárdenas-Morales et al., 2010).

Stroke leads to an imbalance in interhemispheric inhibition, caused initially by a reduced transcallosal activation of contralateral inhibitory networks from the lesioned to the healthy hemispheres (Murase et al., 2004). Reciprocally, the increased excitability of the healthy hemisphere contributes further to the inhibition of the injured hemisphere. rTMS and TBS protocols have been employed with the goal of restoring balance in interhemispheric inhibition. Indeed, cTBS or low frequency rTMS can be used to reduce the over-excitability in the healthy contralateral hemispheric region, and iTBS or high frequency rTMS to increase the excitability of the lesioned cortex (Fitzgerald et al., 2006; Hummel and Cohen, 2006; Chung et al., 2016). These differential effects can be advantageous for people with stroke who tend to overuse their healthy limb. This excessive compensatory use might induce an excitatory reorganization of the contralesional hemisphere and a further increased inhibition of the injured hemisphere, resulting in maladaptive plasticity (Ward and Cohen, 2004). Therefore, stimulation interventions aimed at reducing this interhemispheric imbalance may help stroke patients by limiting maladaptive plasticity and restoring more normal function to the damaged hemisphere (Khan, 2017).

Work in animal models have started to elucidate some of the mechanisms of the TMS modality and TBS protocols specifically in stroke. Sessions of TMS and cTBS over 6 days in rats appears to be neuroprotective for proteins and cell signaling molecules involved in blood brain barrier integrity and promote beneficial changes in angiogenesis and cytokine response (Zong et al., 2020). In mice, cTBS exerts direct and sustained effects via modulation of GABAergic interneuron transmission in photothrombosis (Feng et al., 2020). This is of interest since phasic GABAergic signaling is a potential target for drug therapeutics in mice (Hiu et al., 2016). TMS combined with exercise significantly increased expression of Brain-Derived Neurotrophic Factor and TrkB after middle cerebral artery occlusion in rats (Cui et al., 2020). Hogan and colleagues have written a useful review concerning the molecular mechanisms resulting from neural stimulation and those involving neural plasticity (Hogan et al., 2020). More work in the basic neurosciences will be essential to develop protocols based on an understanding of the molecular processes resulting from neurostimulation which will consequently optimize clinical efficacy.

Although there is promise for rTMS and TBS to improve stroke recovery (Zhang et al., 2017), there are also a number of negative reports from gold-standard randomized control trials (RCTs) (Dionísio et al., 2018; Kim et al., 2020). Several factors likely contribute to this disparity. With regard to patients, there is a lack of consistency in the stroke severity of subjects and the timing of interventions with respect to stroke onset, among other factors known to impact cortical excitability such as age, medications, etc. There is also a lack of consistency in the exact stimulation protocols employed and in the duration over which the effects are monitored post-intervention. This creates a challenge to rigorously assess the therapeutic effectiveness of rTMS and TBS in stroke. While most protocols were effective at inducing changes in the ipsi- and/or contralesional cortical excitability (e.g., Sung et al., 2013; Wang et al., 2014; Du et al., 2016), these changes in excitability were not always correlated to motor improvements (Malcolm et al., 2007; Talelli et al., 2007; Nowak et al., 2008). For many interventions, the induced change in cortical excitability did not translate into long term gains of motor function (Smith and Stinear, 2016). This last point is critical in our opinion: to be effective, stimulation interventions aimed at promoting motor recovery have to be guided by a sound understanding of the precise physiological mechanisms by which neural plasticity can mediate motor recovery, and paired with rigorous validation studies to establish their efficacy. Notably, there are other models of stroke recovery (vicariation, and bimodal balance-recovery utilizing the concept of structural reserve) which rely less on hemispheric imbalance, and may better explain individual variation in therapeutic effectiveness (Di Pino et al., 2014). Further basic science research directly validating these models, along with clinical research in human neuroimaging, will be necessary to determine which perspective is most applicable for a given therapeutic modality.

### Transcranial Direct Current Stimulation (tDCS)

tDCS relies on weak electrical stimulation (e.g., 0.5–4 mA) to modulate cortical excitability. Non-invasive stimulation is applied on the scalp through at least two electrodes, an anode and a cathode, placed above a region or network of interest. It is presumed that cortical excitability is increased in the region under the anode and decreased in the region under the cathode (Nitsche and Paulus, 2001). tDCS is non-invasive and relatively easy to administer, with only potentially mild side effects (Antal et al., 2017). It is also inexpensive relative to other techniques. It can also be set up as a home-based intervention. Its potential as a therapeutic tool has therefore led to a great deal of interest in neuroscience and clinical research.

As mentioned above, stroke may induce an imbalance in interhemispheric inhibition, resulting in maladaptive plasticity. Thus, similar to rTMS or TBS, three main tDCS protocols have been tested in order to restore interhemispheric balance and improve activities of daily living or upper limb rehabilitation (e.g., Plow et al., 2016). The first protocol aims at increasing excitability in the injured region by placing an anode electrode over this region coupled and a cathode electrode on a neutral contralateral region, such as the supraorbital area. A second, inverse strategy is to decrease the excitability of the contralesional cortex by placing the cathode over it, and the anode on the contralateral supraorbital area. Finally, a third approach is to apply both the anode and cathode electrodes over the lesioned and contralesional regions, respectively (Vines et al., 2008). Some early experiments have identified promising effects, such as an hour-long reduction of corticomotor excitability following cathodal stimulation applied over the primary cortex in healthy adults (Nitsche and Paulus, 2001). However, recent randomized studies and meta-analyses of active vs. sham tDCS have found no clear evidence of improvement to upper paretic limb function following any of the tDCS protocols (Bastani and Jaberzadeh, 2012; Triccas et al., 2015; Elsner et al., 2017). With regard to activities of daily living, Elsner et al. (2017) completed a meta-analysis (including 284 participants from 12 randomized controlled trials) and identified a significant moderate effect when the cathode was applied over the contralesional cortex, but no clear evidence for the other protocols.

Nonetheless, in the same manner as rTMS and TBS, tDCS mechanisms are not fully understood yet and the literature still lacks consistency regarding the stimulation parameters used. Some fundamental work has been done in this regard. In mice, anodal high definition tDCS over the contralesional motor cortex after middle cerebral artery occlusion enhanced neurogenesis peri-lesionally and upregulated PDGFA and GDF5 expression in the lesioned hemisphere, factors involved in neuroprotection and downstream plasticity pathways (Ahn et al., 2020). As with TMS, BDNF, TrKB and associated growth factor signaling are heavily implicated as well with tDCS (Fritsch et al., 2010). Although high definition tDCS is still being developed as of writing, and association with functional MRI, magnetic resonance spectroscopy or electroencephalography could be beneficial, the incompleteness of mechanistic understanding hinders the effectiveness of this method. More research is necessary to discover common plasticity mechanisms and pathways across stimulation modalities, thereby identifying high-yield mechanistic targets to optimize stimulation protocols.

### Invasive Electrical Stimulation

Despite the risks associated with surgical implantation of electrodes, invasive neurostimulation techniques have also been investigated. The higher proximity of electrodes to the target neurons confers a superior spatial resolution and stimulation efficiency. These factors may activate and induce plasticity in more targeted neuronal subpopulations, which may in turn provide the means to more effectively remodel neuronal pathways toward functional recovery. For example, epidural stimulation of the perilesional cortex, paired with rehabilitation training, has been tested to promote motor recovery after stroke (Plow and Machado, 2014). Initial experiments in rodents have led to behavioral improvements (Adkins et al., 2008), and Phase I/II clinical trials have also suggested therapeutic benefits (Brown et al., 2006; Levy et al., 2008). Despite the enthusiasm following these results, a Phase III clinical trial unfortunately did not demonstrate significant functional improvement (Levy et al., 2016). This failure could be due to differences in electrode placement among subjects and the diversity of lesion severity, localization and extent (Plow et al., 2009). Further research will be necessary to identify precisely how epidural cortical stimulation could be used to improve motor recovery after stroke.

## Paired Associative Stimulation (PAS)

In PAS paradigms, interventions aim to synchronize perisynaptic neuronal activity to elicit spike-timing-dependent plasticity (Markram et al., 1997; Feldman, 2012). For motor recovery, the most common PAS experiments have combined TMS over the motor cortex and non-invasive electrical stimulation of the spinal cord or peripheral nerves (Stefan et al., 2000; Castel-Lacanal et al., 2007). For example, a study conducted on 19 healthy subjects demonstrated that PAS promoted an increase in primary motor cortex excitability when using transspinal stimulation followed by transcortical stimulation, whereas transcortical stimulation followed by transspinal stimulation induced a decrease in M1 excitability (Dixon et al., 2016). This study also emphasized the importance of interval timing for neuromodulation efficiency. Single sessions of non-invasive PAS in stroke patients are effective at inducing general increases in corticomotor excitability (Palmer et al., 2018). In patients with spinal cord injuries, recent experiments demonstrated that repeated PAS interventions could strengthen the descending connections onto motoneurons and lead to modest but sustained improvements in upper and lower limbs motor function (Bunday and Perez, 2012; Urbin et al., 2017; Jo and Perez, 2020). However, PAS effects are highly variable across subjects (Sale et al., 2007; McGie et al., 2014; Tarri et al., 2018), and failure at inducing plasticity with PAS has also been reported (McGie et al., 2014).

A challenge for PAS (and most rTMS/tDCS) interventions is that stimuli are typically being applied in awake subjects with no regard to the complex ongoing patterns of neuronal activity. The effectiveness of PAS can be increased when stimulation is applied with a specific timing relative to ongoing cortical activity, namely by timing PAS with event-related desynchronization during motor imagery (Royter and Gharabaghi, 2016; Kraus et al., 2018). The effectiveness of this approach underlines the importance of considering the brain’s state and ongoing spontaneous neuronal activity as factors influencing the effectiveness of PAS. Moreover, the effect of PAS has also been shown to be influenced by other processes such as attention (Stefan et al., 2004). Overall, the literature suggests that PAS can be effective, but also that a high level of cooperation between the spontaneous brain activity and stimulation-induced activity is necessary for reliable and effective neuromodulation. The overall idea, namely to administer a neuromodulation treatment during or right after specific states of brain activity, is also important for rTMS treatments: in the Food and Drug Administration (FDA) approved rTMS protocols for smoking cessation or to treat obsessive-compulsive disorders (OCD), subjects are required to observe smoking or OCD symptoms are provoked right before administrating the rTMS treatment, to “activate the relevant neuronal circuits” (FDA, 2018; Roth et al., 2020).

To improve the reliability and efficacy of paired stimulation interventions and advance our understanding of important factors underlying stimulation-induced plasticity, paired stimulation interventions are being investigated in animal models as well. The latter carry inherent advantages over human studies to precisely identify mechanisms and principles of plasticity. For example, at the spinal cord level, coincident stimulation between the descending corticospinal tract and the spinal cord afferents in rats under anaesthesia could induce sustained potentiation of corticospinal excitability (Mishra et al., 2017). An early attempt at invasive electrical PAS in awake rats by our group was not successful in meaningfully potentiating the corticospinal system (Ting et al., 2020). Although many factors could explain this difference, one possible explanation is the interference of spontaneous activity in the very neurons targeted by the pairing process. These PAS results in awake and anesthetized rats contrast with those obtained with rTMS, where interventions on awake animals induced stronger long-term effects that in sedated ones (Gersner et al., 2011). The STDP hypothesis was also tested in awake monkeys using paired stimulation within the sensorimotor cortex, between implanted electrodes, demonstrating that intra-cortical plasticity is also inducible using a paired stimulation protocol (Seeman et al., 2017). However, this study also highlighted inconsistency in PAS outcomes, as robust LTP-like effects were obtained in only 2 out of 15 pairs of cortical sites. Further work in animal models of neurostimulation will be crucial to advance our understanding of the governing principles of plasticity, at both the spinal cord and cortical levels. This process will likely involve more specific methods of neuronal activation, such as optogenetic stimulation in well-defined neuronal subpopulations and circuitry.

## Closed-Loop Stimulation

Therapeutic neuroprostheses rely on healthy brain activity to guide stimulation of the paralyzed limb and produce basic movements such as grasping or reaching. Such closed-loop stimulation paradigms aim to engage more natural patterns of neuronal and muscular activity in the association process. Like paired stimulation targeting cortico-motoneuronal synapses, this approach forces the synchronization between spontaneous (voluntary) corticospinal activity, and stimulation-induced post-synaptic activity (antidromic spikes in motoneurons). Unlike open-loop PAS, however, brain-controlled peripheral stimulation relies on natural patterns of brain activity, and thus engages the very same brain neurons in the association process as those that will be recruited later through voluntary effort. The plastic effects induced through this approach are thus likely to translate more directly into functional gains than open-loop approaches (Ethier et al., 2015).

### Behavior-Controlled Stimulation

A first approach to roughly match neuronal stimulation and ongoing brain activity is to synchronize stimulation to behavior. Promising results have been obtained in severe stroke patients by pairing transcutaneous neurostimulation with rehabilitation exercises (Thrasher et al., 2008). In a series of experiments involving vagus nerve stimulation, closed-loop stimulation of the vagus nerve was found to be effective in driving corticospinal plasticity. For example, Ganzer et al. (2018) demonstrated in rat models of SCI that precisely timed stimulation of the vagus nerve during a rehabilitative isometric pull task could strengthen remaining motor connections. This experiment demonstrated that triggering stimulation in tandem with the most successful movements was significantly better than rehabilitation alone or stimulation associated with weaker pull forces. In rat models of stroke, the same group also demonstrated that vagus nerve stimulation improved functional recovery when paired to rehabilitation exercises, but not when delivered arbitrarily (Khodaparast et al., 2016; Meyers et al., 2018).

An experiment combining electrochemical stimulation and a robotic interface designed to force rats to use their paralyzed hindlimbs has also led to improved recovery of voluntary movement (Van Den Brand et al., 2012). In that study, rats with spinal cord injuries were supported by a harness mounted on a rail and trained to walk on their hindlimbs toward a sweet reward. In this case, the repeated coincidence of voluntary effort and the spinal cord stimulation led to beneficial plasticity and improved recovery. In a related study also in rat models of SCI, stimulation of the spinal cord was triggered upon the detection of residual EMG activity in the impaired forelimb (McPherson et al., 2015). This closed-loop system induced a faster and greater recovery of forelimb function than when the same stimuli were applied with an arbitrary timing or when physical training alone was performed. In all these previous examples, functional benefits were obtained by conjugating stimulation to voluntary effort. This general approach was also successfully tested in human subjects, where the delivery of neuromuscular stimulation during rehabilitation exercises led to increased corticospinal excitability and improved functional recovery (Thrasher and Popovic, 2008; Everaert et al., 2010; Kapadia et al., 2011; Popovic et al., 2011; Hara et al., 2013; Stein et al., 2013). Despite these successes, the strategy of timing stimulation to behavior triggers or EMG recordings has at least two limitations. First, it may be ineffective for patients unable to move, or who display abnormal muscle activity patterns. Second, this approach does not allow for a very high temporal resolution, insufficient perhaps to precisely time the stimulus pulses with respect to action potentials occurring during voluntary effort. Therefore, it may be suboptimal to fully engage Hebbian mechanisms.

### EEG-Controlled Stimulation

Electroencephalography (EEG) is an easy and non-invasive method to record motor cortical activity and infer voluntary effort without relying on movement or EMG activity. Movement intents are correlated to rhythm desynchronization of alpha and beta waves (10–30 Hz) measured over the sensorimotor cortical areas (Neuper and Pfurtscheller, 2001). Brain activity related to both real or imagined movements can be captured in brain-computer interface (BCI) settings to tailor peripheral stimulation to ongoing activity patterns (Iturrate et al., 2018). Controlling functional electrical stimulation using EEG-based BCIs allowed stroke patients to improve upper limb function (Kim et al., 2016; Marquez-Chin et al., 2016; Biasiucci et al., 2018). BCI control of peripheral stimulation via EEGs utilize three main signal sources—the slow cortical potential, sensorimotor rhythms, and event related potentials, specifically the P300 signal. With training, participants can use motor imagery to change the polarity and/or amplitude of one or more of these signals, which enables basic control of computer cursors or other text input devices.

EEG is a convenient method to access spontaneous brain activity and pair it with stimulation in the perspective of rehabilitation, with the primary advantage being that it is non-invasive, and depending on the system, requires varying degrees of initial time investment. However, to reach the scalp electrodes from the brain, the signals have to travel through layers of skin, bone and liquid with different conductivity, inducing distortion of current paths and electrical potentials (Nunez and Srinivasan, 2006). The signals can therefore be contaminated by physiological activity such as spontaneous muscle movements or heart beats (Nunez and Srinivasan, 2006). While positive demonstrations of therapeutic effects were obtained using this approach, EEG recordings also lack the spatiotemporal resolution needed to precisely time stimulation pulses to action potentials or precise patterns of complex neuronal activity. The accuracy that could be obtained with invasive techniques allowing for direct extracellular recordings of neuronal activity (Buzsáki, 2004) could be theoretically advantageous to engage the mechanisms of STDP, requiring the precise detection of action potentials.

### Invasive Recordings and Stimulation

Invasive recording and stimulation techniques are on the rise due to technological advances leading to longer stability and higher signal-to-noise ratios. Unlike EEG, intracortical electrodes enable the detection of action potentials of single neurons with precise temporal and spatial resolution. Intracortical recording is therefore a better candidate than EEG when one wants to induce STDP by precisely controlling the timing of action potentials in different neuronal populations. Similarly, invasive electrodes for intracortical or intraspinal electrical microstimulation can provide a greater spatial precision than magnetic stimulation. Consequently, the use of invasive recording and stimulation electrodes could lead to a more precise intervention, and perhaps a faster and better recovery.

Closed-loop methods using intracortical signals have enabled monkey models and human SCI patients to re-activate and use their own paralyzed muscles voluntarily (Ethier et al., 2012; Bouton et al., 2016; Ajiboye et al., 2017). In addition to enabling movement, closed-loop electrical stimulation has also been applied to drive plasticity in the targeted networks. One important demonstration of this method was made by Jackson et al., who used spike-triggered stimulation within the intact cortex of non-human primates. They were able to induce cortical reorganization which strengthened the functional coupling between distant motor cortical points (Jackson et al., 2006). Nishimura et al. (2013) later demonstrated that both LTP-like and LTD-like plastic effects could be induced in the corticospinal circuits by stimulating monkeys’ spinal cord with different timing after the detection of intracortical spikes. This artificial link between the cortex and spinal cord caused, after 2–3 days of application, a change in the output of the recorded cortical neuron. This was determined using spike-triggered averaging of EMG activity, and this effect lasted for up to a few days. The observed change (LTP or LTD) was consistent with the established STDP causal timing rules.

Few demonstrations of invasive closed-loop systems for brain repair have been documented so far. In an important demonstration, Guggenmos et al. (2013) used a spike-triggered intracortical stimulation approach to strengthen the neuronal connections between the premotor and sensory cortices in rats with a lesion to the primary motor cortex. They demonstrated that this closed-loop approach was more effective than open-loop stimulation to improve functional recovery.

Overall, although different approaches may enable a control over neuronal activity with varying degrees of spatiotemporal resolution, the feature that all closed-loop systems designed to drive plasticity have in common is that they rely on engaging naturally occurring brain activity as the “presynaptic” component of the neuronal association process. The property of such devices relying on spontaneous spiking activity to control stimulation instead of using an arbitrary timing appears to be a defining factor to induce a therapeutically useful reorganization. Therefore, the ability of these systems to engage voluntary efforts might be an important factor for functional recovery (Ethier et al., 2015).

## Leveraging the Selectivity of Optogenetics to Study and Guide Plasticity Toward Rehabilitation

Regardless of their spatial resolution, electrical and electromagnetic neurostimulation methods activate nearby neuronal elements indiscriminately of their phenotype or projection targets. Optogenetic techniques may provide an important theoretical advantage over electrical and magnetic stimulation in that respect, as opsins can be virally expressed in cells with specific genetic signatures or innervation patterns, thanks to genetic promoters and conditional or intersectional approaches (Fenno et al., 2011). Additionally, when targeting nerves or muscles, electrical stimuli do not recruit muscle fibers according to Henneman’s size principle (Henneman et al., 1965), but instead tends to activate small and large fibers unselectively (Gregory and Bickel, 2005). Such a recruitment pattern can lead to quicker muscle fatigue, a problem that is alleviated with optogenetic nerve stimulation, which leads to a more natural order of motor unit recruitment (Llewellyn et al., 2010; Williams et al., 2019). Optogenetic stimulation can therefore activate neurons with more natural activity patterns, with a timing resolution of milliseconds, and with the cellular specificity of pharmacological methods (Fenno et al., 2011), permitting the selective control of neurons with specific genetic identities or projection targets within a given volume of brain tissue.

### Central Optogenetic Stimulation

Optogenetic stimulation may provide more consistent neuromodulation results than electromagnetic stimulation. Wu et al., conducted iTBS and cTBS experiments using optical stimulation in rodent M1. In contrast to electromagnetic stimulation, their optical stimulation targeted excitatory cells selectively and not inhibitory interneurons. As a result, both iTBS and cTBS protocols induced a potentiation of the corticospinal projections (Wu et al., 2018). Importantly, they also reported that optogenetic iTBS led to LTP-like effects which were nearly twice as strong as when iTBS was delivered electrically. The selectivity achievable with optogenetic tools therefore seems to allow an important increase in effectiveness in inducing plasticity. Yazdan-Shahmorad et al. (2018) investigated optically-induced cortical reorganization inside macaques’ sensorimotor cortex. Using a large scale optogenetic interface, they demonstrated that functional connectivity between the motor and somatosensory cortex could be strengthened following a Hebbian plasticity model (Yazdan-Shahmorad et al., 2018). Provided that optogenetic technologies evolve to a point where they can be safely administered in humans, there is a huge potential for enhanced rehabilitation protocols using selective optical neuronal stimulation.

### Peripheral Optogenetic Stimulation

In comparison to electrical stimulation, optogenetic stimulation allows for a more orderly motor unit recruitment pattern which more closely resembles a physiological contraction (Llewellyn et al., 2010). To test this characteristic, Srinivasan et al. (2018) compared closed-loop functional optical stimulation (FOS) and functional electrical stimulation (FES) on mice and rats’ peripheral nerves to control ankle joint position. The closed-loop system combined optical or electrical stimulation to a distance sensor measuring ankle joint position as feedback (Srinivasan et al., 2018). Results showed that FOS was more accurate and presented faster rise times than FES. Also, FES induced more fatigue during periodic movements, indicating that optogenetic methods may be better suited for longer and/or more repetitive use. Although early tests showed limited time course of effectiveness, virally-mediated optogenetic activation of peripheral nerves have been successfully tested in non-human primates as well (Williams et al., 2019).

Before developing fully optical closed-loop devices, it may be possible to combine electrical monitoring and optical stimulation in a hybrid device (Song et al., 2018). In their study, Song et al. (2018), developed a nerve cuff combining platinum electrodes and a blue light emitting diode to record and stimulate the peripheral nerves of active Thy1:ChR2 transgenic mice. Efficient light penetration deep into peripheral neural tissue renders optogenetic stimulation less position dependent than electrical stimulation without losing specificity. The validation of transdermal optical stimulation (Srinivasan et al., 2018) could make non-invasive optogenetic methods feasible in the near future. Development of these technologies would eliminate the biological risks inherently associated with implants and invasive surgeries and may facilitate the clinical transition of optogenetics.

### Limitations of Optogenetics

Closed-loop optogenetic stimulation in animal models have delivered promising results for the development of innovative and efficient rehabilitation devices. However, further progress with optogenetic technologies and molecular tools will be necessary to achieve an efficient transition from animal experiments to human clinical treatment.

One of the core challenges to be addressed concerns opsin stimulation efficiency. When experimenting on non-human primates, devices which induced promising results on rodent brains would have to tackle a volume approximately 100 times larger (Herculano-Houzel, 2009). This volume scaling might induce more light scattering and therefore reduce opsin stimulation intensity. One might increase the optic fiber’s diameter, but this could produce more tissue damage (Diester et al., 2011). On the other hand, the use of above-the-surface optical fiber or external brain illumination could reduce brain damage, but might not be efficient for deep brain tissues (Ruiz et al., 2013). Some new light emitting devices already address these limitations, such as large-volume illuminators, which are optical fibers modified to obtain an emitting surface area 100 times larger than conventional fibers (Acker et al., 2016). The development of red-shifted opsins, activated by light with longer wavelengths such as ChrimsonR (excitatory) (Klapoetke et al., 2014) or Jaws (inhibitory) (Chuong et al., 2014), may enable stimulation of deeper and larger structures. For example, large volume illuminators combined to Jaws in the rhesus monkey frontal eye field has induced large behavioral changes and inactivation of 80–100% neurons over 10 mm^3^ (Acker et al., 2016). Similar studies with conventional fibers reported 38–68% of neuron inactivation within 1 mm of the light source (Diester et al., 2011; Han et al., 2011).

An additional challenge concerns effective gene transfer to human cells. Adeno-associated viruses (AAVs), are an efficient tool to transduce opsin coding genes to cells. AAVs are valuable carriers due to their neural tropism as well as their ability to transport opsin genes and cell-specific promoters (Montgomery et al., 2016). Although effective, this technique should be used carefully to prevent immunogenic effects due to high viral doses inside cells. Similar dose-dependent immune responses have been observed in a clinical trial for degenerative retinal disease using AAVs (Bainbridge et al., 2015). Cellular transplantation of opsin-expressing cells is an alternative to AAV usage, as transplantation of autologous cells could limit immune responses. This method was proven effective to modulate muscle activity in mice suffering from partial denervation (Bryson et al., 2014). Further research, and eventually human trials, are required in this area to demonstrate feasibility and safety.

## Neuromodulation Beyond Neurostimulation: Other Factors Influencing Neural Plasticity

There are other very promising research directions which are being investigated for harnessing beneficial plasticity through neurotechnologies. The critical advances will come from a better fundamental understanding of systems neuroscience and mechanisms of neural plasticity. Perineuronal nets are neuroprotective extracellular matrix formations which have demonstrated influence on synaptic plasticity through modulation of lateral AMPAR diffusion during critical periods (Testa et al., 2019). Nogo-A and NgR1 signaling is also a potentially important plasticity-limiting mechanism, and early work has demonstrated that anti-Nogo antibody/NgR1 receptor antagonists may be helpful to induce new periods of experience-dependent plasticity, for diseases ranging from spinal cord injury, to stroke and multiple sclerosis (Schwab and Strittmatter, 2014). Finally, neuromodulators such as dopamine and serotonin may play essential roles in controlling when and how plasticity is expressed. We know that dopamine signaling, for example, is a necessary component of STDP-type plasticity in the corticostriatal system (Pawlak and Kerr, 2008; Yagishita et al., 2014).

The ideal therapeutic treatment will likely be multi-modal and evidence-based, involving not only optimized timing (closed-loop) and specificity (optogenetics), but also effective neuromodulation using reward cues and/or pharmaceuticals to create a fertile environment for beneficial training-induced neural plasticity. It is also critical to understand that stroke is not a homogeneous phenomenon across patients. Every neurovascular event is unique in terms of location, severity and affected systems. With that in mind, it is crucial that future therapeutic interventions be tailored to each individual patient and their specific needs. Indeed, that is part of the challenge. However, with all the emerging knowledge on neural plasticity and improvement in stimulation techniques and protocols, it is likely that the upcoming decades will lead us to the emergence of highly effective interventions to drive neuronal reorganization. Severe neurological insult may no longer be synonymous with lifelong impairment.

## Conclusion

In this review, we posit that simultaneous advances along several fronts will be necessary to create more effective therapeutic stimulation interventions, including but not limited to closed-loop approaches and the integration of optogenetics. Fundamental research into the rules and governing principles of plasticity will be critical to this endeavor. We may be at the cusp of a revolution in rehabilitation neuroscience, where our understanding of the brain and neuronal plasticity can be directly applied to help patients recover from neurological insult.

## Author Contributions

WT, FF, and CE designed the review. WT, FF, SF, and CE wrote the manuscript. All authors contributed to the article and approved the submitted version.

## Conflict of Interest

The authors declare that the research was conducted in the absence of any commercial or financial relationships that could be construed as a potential conflict of interest.
